# Sustained morphine exposure alters spinal NMDA receptor and astrocyte expression and exacerbates chronic pain behavior in female rats

**DOI:** 10.1097/PR9.0000000000001145

**Published:** 2024-03-12

**Authors:** Sara V. Gonçalves, Stephen G. Woodhams, Li Li, Gareth J. Hathway, Victoria Chapman

**Affiliations:** a Pain Centre Versus Arthritis, University of Nottingham, Medical School, Queen's Medical Centre, Nottingham, United Kingdom; b School of Life Sciences, Medical School, Queen's Medical Centre, Nottingham, United Kingdom; c NIHR Nottingham Biomedical Research Centre, University of Nottingham, Nottingham, United Kingdom

**Keywords:** Opioids, NMDA receptors, Astrocytes, Females, Chronic pain

## Abstract

**Introduction:**

Sustained opioid use has long-term negative impacts on future pain experience, particularly in women. This study aimed to investigate the underlying spinal neurobiology of this clinical observation in an experimental model of joint pain.

**Objectives:**

In this study, we tested the hypothesis that sustained opioid treatment exacerbates chronic pain responses and alters spinal cord dorsal horn astrogliosis and the expression of GluN2B-containing N-methyl-d-aspartate receptors in female rats.

**Methods:**

Subcutaneous morphine (3 mg/kg) or saline was administered twice daily for 1 week before inducing a model of joint knee pain (intra-articular injection of 2 mg of monosodium iodoacetate [MIA]) in adult female Sprague-Dawley rats, with pain-free controls receiving 50 µL of saline. Pain behavior (weight-bearing and mechanical paw withdrawal thresholds) was measured at baseline and at intervals thereafter. Twice-daily morphine/saline treatment was continued for up to 3 weeks after intra-articular injections, and spinal cord tissue was collected for Western blot analyses.

**Results:**

Area under the curve analysis of weight-bearing asymmetry confirmed a significant exacerbation of pain behavior in the morphine/MIA group, compared with the saline/MIA group (F_(3,18)_ = 46.3, *P* < 0.0001), despite comparable joint damage in both groups. Sustained morphine treatment was associated with significant elevations in dorsal horn expression of astrocytic glial fibrillary acidic protein (27 ± 5% increase) and neuronal GluN2B (80 ± 30% increase), but not microglial IBA1, irrespective of the model of joint pain.

**Conclusion:**

These data suggest that sustained morphine treatment in female rats drives spinal cord plasticity, including spinal astrogliosis and the expression of GluN2B-containing N-methyl-d-aspartate receptors, priming the dorsal horn to incoming sensory inputs and producing exacerbated pain responses.

## Abstract 2

Supplemental Digital Content is Available in the Text.

Sustained morphine treatment increases subsequent osteoarthritis-like pain in female rats, mimicking the clinical picture. Spinal synaptic plasticity and astrogliosis are implicated as potential mechanisms.

## 1. Introduction

Persistent pain affects millions of lives globally, leaving a long-term legacy of negative emotional, societal, and economic consequences.^45^ The impact is greatest on female individuals, who are more likely to report chronic pain and are prescribed more opiate analgesics than male individuals.^1,50^ Despite this, most of our experimental knowledge of chronic pain and opioid receptor mechanisms is derived from studies of male rodents.^36^

Chronic pain states are associated with increased pain responses (hyperalgesia), arising from a combination of peripheral sensitization and plasticity of the spinal cord dorsal horn (central sensitization),^26^ involving changes in neuronal and immune cell signaling. Both neuroinflammatory signaling and endogenous pain control mechanisms contribute to central sensitization^19,34^ in a sexually dimorphic manner.^12^ N-methyl-d-aspartate receptors (NMDARs) have pivotal roles in hyperalgesia.^13,27^ Both GluN2A-containing and GluN2B-containing NMDARs are present in the spinal cord dorsal horn,^38^ with GluN2B-containing NMDARs particularly implicated in chronic pain in animal models.^15,23,43^ GluN2B-containing NMDARs bidirectionally regulate synaptic plasticity in the hippocampus and can drive destabilization of memories (see references in^48^). Similarly, nonionotropic NMDARs have a role in reactive desensitization of dorsal horn excitability, suggesting synaptic destabilization in spinal cord circuitry may play a role in central sensitization and pain memory reconsolidation.^55^

Opioids are powerful short-term analgesics, but converging evidence suggests that prior opioid use worsens outcomes to subsequent painful injury.^19,52^ Prolonged opioid use can cause opioid-induced hyperalgesia and worsen pain trajectories.^9,46^ Opioid use before joint replacement in osteoarthritis (OA) was associated with increased postoperative pain,^9^ increased opioid consumption, and delayed postoperative pain resolution.^39,57^ Opioid use on the day of surgery was associated with greater opioid use at 6 months.^17^ Experimentally, repeated exposure to µ-opioid receptor (MOR) ligands sensitizes primary afferent sensory fibers to subsequent acute noxious stimuli,^3,46^ and repeated morphine treatment following induction of a model of chronic pain increased spinal expression of markers of both microglia and astrocytes in rodents.^44^ Morphine tolerance is associated with decreased spinal cord expression of glutamate transporters.^22,31^ Morphine-induced mechanical hypersensitivity is dependent on β-arrestin2,^51^ which also regulates mechanical allodynia and GluN2B-mediated NMDAR currents in spinal cord neurons.^8^ Whether sustained morphine treatment alters spinal GluN2B expression is unknown.

This study aimed to advance mechanistic understanding of the effects of long-term opioid treatment on the spinal cord neurobiology of pain processing in a translationally relevant model. In this study, we back-translate clinical evidence that sustained opioid treatment exacerbates future pain experience in a model of OA-like joint pain in female rats. We test the hypothesis that dorsal horn sensitization mechanisms, specifically astrogliosis and altered expression of GluN2B-containing NMDARs, are associated with the detrimental effects of sustained opioid treatment on joint pain in this model.

## 2. Methods

### 2.1. Experimental animals

**Figure 1.**
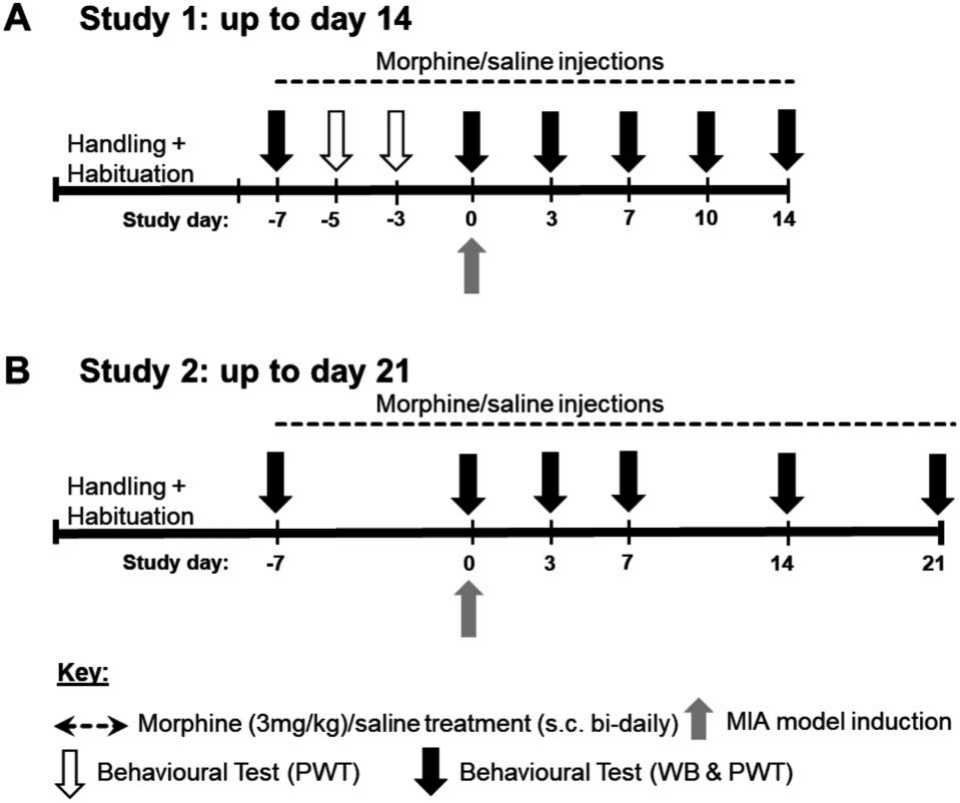
Overview of experimental timeline: The effect of pre-exposure to morphine on OA-like pain behavior. (A) Study 1: Female SD rats received bi-daily dosing with systemic morphine (3 mg/kg, s.c.) or saline for 1 week before intra-articular injection of 2 mg MIA or saline. Systemic treatment with saline or morphine continued for a further 2 weeks. Pain behavior was assessed 1 hour before and after morning injection of morphine or saline to provide measures of both chronic and acute effects of treatment. (B) Study 2: Female SD rats received bi-daily dosing with systemic morphine (3 mg/kg, s.c.) or saline for 1 week before intra-articular injection of 2 mg MIA or saline. Systemic treatment with saline or morphine continued for a further 3 weeks (n = 6/group/study: saline/saline, saline/MIA, morphine/saline, and saline/MIA). To focus on the chronic effects of morphine, behavioral assessments were made 1 hour before the first dose each morning. MIA, monosodium iodoacetate; OA, osteoarthritis; PWTs, paw withdrawal thresholds; s.c., subcutaneous; SD, Sprague-Dawley; WB, weight-bearing.

Two studies (Fig. 1) were run using separate cohorts of 24 young adult female Sprague-Dawley rats (total n = 48, 175–200 g, ∼8 weeks old at arrival, Charles River, United Kingdom). Rats were housed 4/cage in double-decker, individually ventilated cages under temperature-controlled and humidity-controlled conditions (21 ± 1.5°C, 50 ± 8%) with alternating 12-hour light/dark cycle (lights on at 7 am) and access to food and water ad libitum. Rats acclimatized to the holding facility for 3 to 7 days on arrival and were extensively handled to habituate animals the experimenter before behavioral testing. Study 1 was performed by a female experimenter and study 2 by a male experimenter, and all behavioral tests were conducted during the light phase. Studies were approved by the University of Nottingham's Animal Welfare and Ethical Review Board (AWERB) in accordance with the UK Home Office Animals Scientific Procedures Act (1986) and the International Association for the Study of Pain guidelines. Procedures are reported in compliance with the ARRIVE 2.0 guidelines.^42^ One rat (morphine/MIA) in study 1 was excluded from weight-bearing analysis due to signs of anxiety and hyperactivity preventing reliable behavioral data collection.

### 2.2. Modelling sustained opioid exposure in female rats

Morphine hydrochloride (3 mg/kg, Bio-Techne, Abingdon, United Kingdom) or saline (1 mL/kg) was injected subcutaneously (s.c.), twice daily (∼9 am and 4 pm). Treatment began 1 week before model induction and continued until study termination. Morphine dose and schedule were based on previous studies,^14,37^ and 3 mg/kg s.c. morphine significantly reverses neuropathic pain behavior without affecting motor performance.^37^

### 2.3. Induction of the monosodium iodoacetate model of osteoarthritis joint pain

After 7 days of pretreatment with systemic morphine or saline, rats received a single intra-articular injection of either 2 mg/50 µL of monosodium iodoacetate (MIA; Sigma, United Kingdom) to model OA-like knee joint pain or 50 µL of 0.9% saline (control group). Injections were administered through the infrapatellar ligament of the left knee using a 30-gauge needle under brief isoflurane anesthesia (3%, 1L/min O2), as previously described.^28^ Each study used 4 treatment groups (n = 6/group): 1. Saline/saline—opioid-naïve, pain-free controls; 2. saline/MIA—opioid-naïve with OA-like pain; 3. morphine/saline—sustained opioid exposure, pain-free; and 4. morphine/MIA—sustained opioid exposure with OA-like pain.

The experimental unit was an individual rat. Rats were randomized to treatment groups through a random number generator, with 1 animal/group allocated to each cage to minimize potential environmental confounders. Experimenters were blinded to treatment throughout data collection and analysis. Welfare checks were performed immediately after anesthetic recovery, daily for 5 days, and weekly thereafter. Humane end points were >10% weight loss in 3 days, or 20% overall, unprovoked vocalization lasting >10 seconds, joint swelling >200% of baseline, failure to feed or drink, starey coat or persistent grimace, or excessively impaired movement. No animals exceeded these limits. The MIA dose was based on previous studies^25,40^ and on a pilot study (Figure S1, http://links.lww.com/PR9/A224). Intra-articular injection of 2 mg/50 µL of MIA produced robust pain behavior and OA-like knee pathology in young adult female Sprague-Dawley rats, while a 1 mg dose produced variable joint pathology without significant pain behavior. These data were used to calculate group sizes.

### 2.4. Behavioral testing

Behavioral measurements were made on specific days for 2 (study 1, Fig. 1A) or 3 (study 2, Fig. 1B) weeks after intra-articular injections. Measurements were collected 1 hour before morning injection to assess chronic effects of treatment and 1 hour after morning injection in study 1 only (Fig. 1A) to evaluate acute effects. Weight-bearing (WB) asymmetry was assessed using an incapacitance tester (Linton Instrumentation, Diss, United Kingdom),^5^ and absolute mechanical paw withdrawal thresholds (PWTs) were determined for both hind paws through the up/down method.^7,10^ Weight-bearing asymmetry was calculated as % [ipsilateral g/(ipsilateral g + contralateral g)]. Because intervals between successive von Frey hairs are logarithmic, PWTs were reported as log(g).^35^

### 2.5. Tissue collections

Animals were killed at study end through anaesthetic overdose (sodium pentobarbital, 2 mL, i.p.) 1 hour after morning dosing, and fresh spinal cord tissue was collected. Lumbar (L1-L6) spinal cords were split into quadrants, snap frozen in liquid nitrogen, and stored at −80°C until analyses. Knee joints were dissected and fixed in 10% neutral-buffered formalin for assessment of joint pathology.

### 2.6. Assessment of joint pathology

Knee joints were disarticulated, and cartilage damage was scored macroscopically by 2 independent, blinded experimenters, as previously described.^20^ In brief, chondropathy was scored in 5 different compartments (medial tibial plateau, lateral tibial plateau, medial femoral condyle, lateral femoral condyle, and femoral groove) using a 5-point scoring system (0 = normal appearance; 1 = slight yellowish discoloration of the chondral surface; 2 = little cartilage erosions in load-bearing areas; 3 = large erosions extending down to the subchondral bone; and 4 = large erosions with large areas of subchondral bone exposure). Scores were summed to determine aggregate total chondropathy for each knee joint on a scale of 0 to 20.

### 2.7. Protein isolation and Western blotting

Lumbar (L1-L6) spinal ipsilateral dorsal horn quadrants were homogenized in RIPA buffer (50 mM Tris hydrochloride pH 7.4, 150 mM sodium chloride, 1% Triton X-100, 0.5% sodium deoxycholate, 0.1% SDS, 1 mM ethylenediaminetetraacetic acid (EDTA), EDTA-free complete protease inhibitor cocktail, Roche, Basel, Switzerland), placed on ice for 2 hours and cleared by centrifugation. Protein concentration curves were run for each antibody and target protein combination to determine the saturation point of densitometry and select appropriate sample loading volumes. Lysate was denatured in Laemmli buffer (100°C, 5 minutes), proteins were separated by electrophoresis on 9% to 10% polyacrylamide SDS-PAGE gel, transferred to a nitrocellulose blotting membrane (GE Healthcare, Chicago, IL), and blocked in 5% Marvel. Membranes were incubated with primary antibodies overnight at 4°C (ionized calcium-binding adapter molecule 1 [IBA1]; glial fibrillary acidic protein [GFAP]; GluN1; GluN2B; and β-actin; see Table S1, http://links.lww.com/PR9/A224 for full details). Antibody binding was visualized using 680 or 800 nm dye-conjugated donkey anti-rabbit and goat anti-mouse secondary antibodies (1:10000; LiCor, Lincoln, NE) and detected through LiCor Odyssey Imaging System (LiCor). Protein expression relative to β-actin was quantified by densitometry using Image Studio Lit v5.2 software.

### 2.8. Statistical analyses

Data were tested for normality using D'Agostino-Pearson or Shapiro-Wilk tests and analyzed through parametric or nonparametric tests, as appropriate, using GraphPad Prism 9.5.1. Weight-bearing and PWT data were analyzed using analysis of variance (ANOVA) with group as between-subjects factor and testing day as repeated measures/within-subjects factor (RM 2-way ANOVA).

Longitudinal changes in pain phenotypes in experimental models are highly amenable to area under the curve (AUC) analysis of the full time course. Area under the curve analyses were assessed through 1-way ANOVA with the Holm-Šídák multiple comparison test or the Kruskal-Wallis test with the Dunn multiple comparison test where normality assumption was not met. Cartilage damage scores and relative protein expression were analyzed through 2-way ANOVA with the Tukey multiple comparison test, with drug treatment and model as independent variables, or the Kruskal-Wallis test with the Dunn multiple comparison test. Percentage change was calculated as follows: (AUC mean group X − AUC mean Y)/AUC mean group Y × 100. To explore effects of drug treatment on the relationship between pain behavior and spinal protein expression, correlation analyses were performed. Data from each drug treatment group (morphine naïve = saline/saline and saline/MIA, morphine-treated = morphine/saline & morphine/MIA) were pooled, and correlations between pain behavior (PWT AUC & WB AUC) and spinal protein expression were determined through 1-tailed Pearson *r*. *P* < 0.05 was accepted as significant for all analyses.

## 3. Results

### 3.1. Sustained exposure to morphine exacerbates pain behavior in a model of joint pain

**Figure 2.**
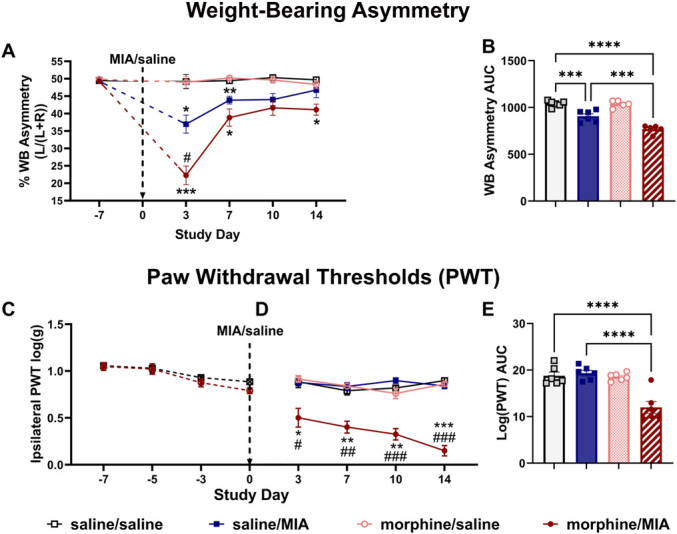
Sustained exposure to morphine is associated with greater MIA-induced pain behaviour in female rats. See Figure 1A for study design and timeline. (A) Weight-bearing asymmetry was observed following unilateral intra-articular injection of MIA in all rats, but to a significantly greater degree in rats treated with morphine. (B) Area under the curve (AUC) analysis of the time course data in (A) confirmed that morphine treatment exacerbated weight-bearing asymmetry when compared with saline treatment in the MIA model (saline/saline = 6, saline/MIA = 6, morphine/saline = 6, and morphine/MIA = 5). (C) Neither systemic administration of saline nor morphine altered PWTs before model induction (morphine/naïve = 12 and saline/naïve = 12). (D) Following intra-articular injection of 2 mg MIA, PWTs were robustly decreased in rats treated with morphine, but not those treated with saline. (E) AUC of the time course data in (D) confirmed no change in PWTs in either the morphine/saline and saline/MIA groups compared with saline/saline group, but a significant decrease in PWTs in the morphine/MIA–treated group (n = 6 for all groups). Data are presented as mean ± SEM. *P = 0.5, **P < 0.001, and ***P < 0.0001 vs morphine/saline; #*P* < 0.05, ##*P* < 0.001 vs saline/MIA. RM 2-way ANOVA with the Tukey *post hoc* testing. ANOVA, analysis of variance; MIA, monosodium iodoacetate; PWT, paw withdrawal threshold.

To quantify joint pain following intra-articular injection of MIA in female rats, 2 measures of pain behavior were assessed for 14 days. Morphine-naïve rats (saline/MIA) exhibited significant WB asymmetry at D3-D7, but not later time points (Fig. 2A). By contrast, the morphine-treated joint pain group (morphine/MIA) exhibited significantly greater WB asymmetry at D3, which was maintained until D14 (Fig. 2A). Area under the curve analysis confirmed significant exacerbation of WB asymmetry in morphine/MIA compared with saline/MIA (morphine/MIA = 768 ± 20 saline/MIA = 906 ± 25, mean ± SEM; F_(3,18)_ = 41.5, *P* < 0.0001, Fig. 2B). Female rats exhibited marginal lowering of PWTs over the week before model induction, irrespective of the treatment group (Fig. 2C; group: F_(1,22)_ = 4.5, *P* = 0.34; time: F_(2.4,52.3)_ = 25.0, *P* < 0.0001; time × group: F_(3,66)_ = 1.04, *P* = 0.38). Following induction of joint pain, the morphine/MIA group exhibited profound and sustained lowering of PWTs for the duration of the study (Fig. 2C, D; group: F_(3,20)_ = 49.1, *P* < 0.0001; time: F_(2.6,52.6)_ = 6.4, *P* = 0.0008; time × group: F_(9,60)_ = 4.75, *P* < 0.0001). By contrast, PWTs were unaltered in the saline/MIA group. Area under the curve analyses confirmed significant exacerbation of PWT lowering in morphine/MIA compared with both saline/saline and saline/MIA groups (Fig. 2E; morphine/MIA = 12.0 ± 1.3 vs saline/saline = 18.8 ± 0.8 and saline/MIA = 18.6 [17.8–19.2]; F_(3,20)_ = 17.4, *P* < 0.0001).

Our study design also probed the acute analgesic effect of morphine and the development of morphine tolerance. On D3 in the joint pain model, WB asymmetry was significantly attenuated 1 hour post morphine (supplementary Figure S2A, http://links.lww.com/PR9/A224). This inhibitory effect was not seen on later days (supplementary Figure S2A, http://links.lww.com/PR9/A224), indicative of analgesic tolerance to morphine. Morphine treatment also partially reversed lowered PWTs throughout the study duration (supplementary Figure S2B, http://links.lww.com/PR9/A224).

**Figure 3.**
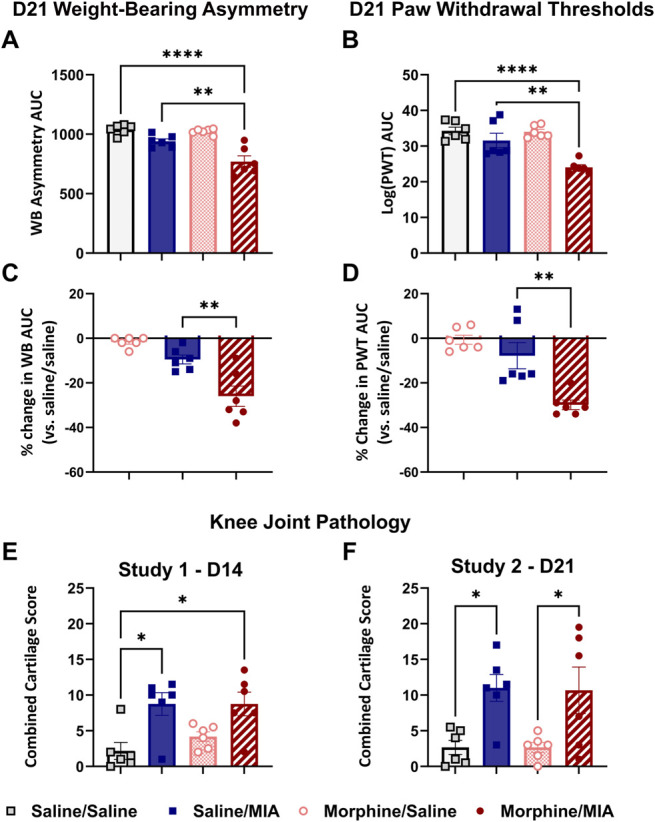
Morphine-induced exacerbation of pain behaviour is not accompanied by greater joint pathology at 14 or 21 days after model induction A. AUC analysis of pain behaviour time course from study 2 (see Fig. 1B for timeline) confirmed the morphine-induced exacerbation of MIA-induced changes in weight-bearing asymmetry (A and C) and PWTs (B and D) at day 21. % change in weight-bearing asymmetry or log(PWTs) was calculated by comparing AUC data from each group with saline/saline–treated animals, with 0% representing no difference in value. Data are presented as mean ± SEM, n = 6/group. **P < 0.01, ****P < 0.0001. One-way ANOVA with the Tukey multiple comparison *post hoc* test. (E). At D14, mean combined cartilage damage scores were higher for both the saline/MIA and morphine/MIA groups compared with the saline/saline group, *P < 0.05 vs saline/saline, Kruskal-Wallis test with the Dunn multiple comparison *post hoc* test. (F) Similarly, mean combined cartilage damage scores were higher for both the saline/MIA and morphine/MIA groups compared with the saline/saline and morphine/saline groups at D21 (*P* < 0.05 vs saline-treated controls, 2-way ANOVA with the Tukey multiple comparison *post hoc* test), with no significant difference between saline/MIA and morphine/MIA at either D14 or D21. ANOVA, analysis of variance; AUC, area under the curve; MIA, monosodium iodoacetate; PWT, paw withdrawal threshold.

Spinal features of central sensitization are present at later time points (D21 onwards) in this joint pain model.^49^ To probe potential contributions of these mechanisms to the morphine-induced exacerbation of pain behavior observed, a replication study was performed with an extended timeline (study 2, D21). Comparison of AUC data for WB asymmetry and PWT up to D14 revealed concordance between studies 1 and 2 (supplementary Figure S3, http://links.lww.com/PR9/A224), demonstrating that the augmented pain phenotype seen with sustained morphine treatment and the model of joint pain is highly reproducible. At D21 after model induction, AUC analysis confirmed significant WB asymmetry (Fig. 3A; morphine/MIA = 770 ± 48, vs saline/saline = 1040 ± 17, saline/MIA = 940 ± 21; F_(3,20)_ = 19.35, *P* = 0.0019 and *P* < 0.0001) and lowered PWTs in the morphine/MIA group (Fig. 3B; morphine/MIA = 24.0 ± 0.7, vs saline/saline = 34.3 ± 1.1, saline/MIA = 31.6 ± 2.0; F_(3,20)_ = 14.70, *P* = 0.0019 and *P* < 0.0001). Analysis of % change in WB (Fig. 3C; morphine/MIA = −26.0 ± 4.6%, vs saline/MIA = −9.5 ± 2.0; F_(2,15)_ = 18.01, *P* = 0.0032) and PWTs (Fig. 3D; morphine/MIA = 30.0 ± 2.1% vs saline/MIA = −7.8 ± 5.9%, F_(2,15)_ = 16.13, *P* = 0.0002) confirmed morphine-induced exacerbated joint pain in this model at D21.

### 3.2. Comparable monosodium iodoacetate–induced joint pathology in the presence and absence of morphine

To determine whether morphine-induced exacerbation of pain arises due to differences in joint pathology, gross cartilage damage was scored macroscopically. At both D14 and D21 after model induction, MIA induced significant cartilage damage compared with intra-articular injection of saline (Fig. 3E, F). At D14, total cartilage damage scores were as follows: saline/saline = 1.25 [0.38–3.5] vs saline/MIA = 10 [7–11.5], *P* = 0.04; morphine/saline = 4.5 [2.38–5.63]; and morphine/MIA = 9 [6.13–12], *P* = 0.03, median [IQR]; Kruskal-Wallis test with the Dunn multiple comparison test. At D21, total cartilage damage scores were as follows: saline/saline = 2.67 ± 1.00; saline/MIA = 11.00 ± 1.89, *P* < 0.05 vs saline/saline and saline morphine; morphine/saline = 2.67 ± 0.70; and morphine/MIA = 10.67 ± 3.27, mean ± SEM, *P* < 0.05 vs saline/saline and saline morphine, 2-way ANOVA with the Tukey multiple comparison test. Of importance, morphine treatment did not alter the magnitude of cartilage damage in this model.

### 3.3. Sustained morphine exposure upregulates a spinal marker for astrocytes, but not microglia

**Figure 4.**
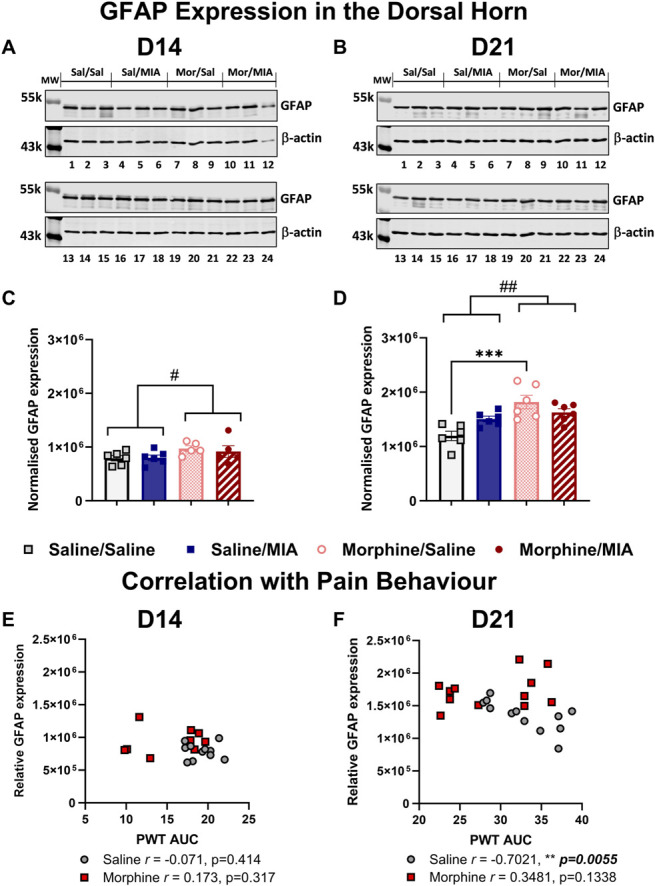
Sustained exposure to morphine upregulates GFAP expression in the dorsal horn of the spinal cord in female rats and uncouples GFAP expression from OA-like pain behaviour. GFAP protein expression was quantified in the ipsilateral spinal cord dorsal horn at D14 (A) and D21 (B) after intra-articular injection. See supplementary Figure S4, http://links.lww.com/PR9/A224 for full blots. Densitometry of GFAP labelling intensity expressed relative to β-actin in arbitrary units at D14 (C) and D21 (D). Morphine-treated groups had significantly elevated GFAP expression at D14 after intra-articular injection; there was no effect of the MIA model at this time point (n = 5–6/group). Similarly at D21, morphine treatment was also associated with significantly elevated GFAP expression (n = 6/group) irrespective of the presence of OA-like pain. Data are presented as mean ± SEM and analysed through 2-way ANOVA with the Tukey multiple comparison *post hoc* test. #*P* < 0.05, #*P* < 0.01 main effect of drug treatment, *P < 0.05, ***P < 0.001 multiple comparisons between groups. There was no relationship between PWT AUC values and spinal GFAP expression at D14 (E) in either saline-treated or morphine-treated rats. At D21 (F), PWTs were negatively correlated with GFAP expression in the morphine naïve groups but not in the morphine-treated groups (data pooled by drug treatment and correlations determined through Pearson correlation coefficient *r*). ANOVA, analysis of variance; GFAP, glial fibrillary acidic protein; MIA, monosodium iodoacetate; OA, osteoarthritis; PWT, paw withdrawal threshold.

Expression of GFAP and IBA1, markers for astrocytes and microglia, respectively, were quantified in the ipsilateral spinal dorsal horn. GFAP expression was quantified at D14 (Fig. 4A) and D21 (Fig. 4B) after intra-articular injection of saline or MIA in the presence or absence of morphine treatment. There was a significant effect of drug treatment (F_(1,18)_ = 4.80, *P* = 0.04), but not model (F_(1,18)_ = 0.12, *P* = 0.73), with morphine treatment increasing the expression of GFAP at D14 in both groups (Fig. 4C; saline/saline = 791667 ± 49752, saline/MIA = 800946 ± 51471, morphine/saline = 971703 ± 52728, and morphine/MIA = 915500 ± 108177, mean ± SEM). GFAP expression was significantly elevated in both morphine-treated groups when compared with saline-treated groups at D21 (Fig. 4D; saline/saline = 1195326 ± 85797, saline/MIA = 1506462 ± 51891, morphine/saline = 1817537 ± 123836, morphine/MIA = 1625229 ± 71038, mean ± SEM), again there was no effect of the model of joint pain (F_(1,20)_ = 0.46, *P* = 0.50). At D21, effects of morphine on GFAP expression were greater compared with those at D14 (F_(1,20)_ = 18.04, *P* = 0.0004). No significant differences in IBA1 expression were observed between any of the groups at D21 (supplementary Figure S5, http://links.lww.com/PR9/A224).

### 3.4. Sustained morphine treatment upregulates spinal expression of GluN2B

**Figure 5.**
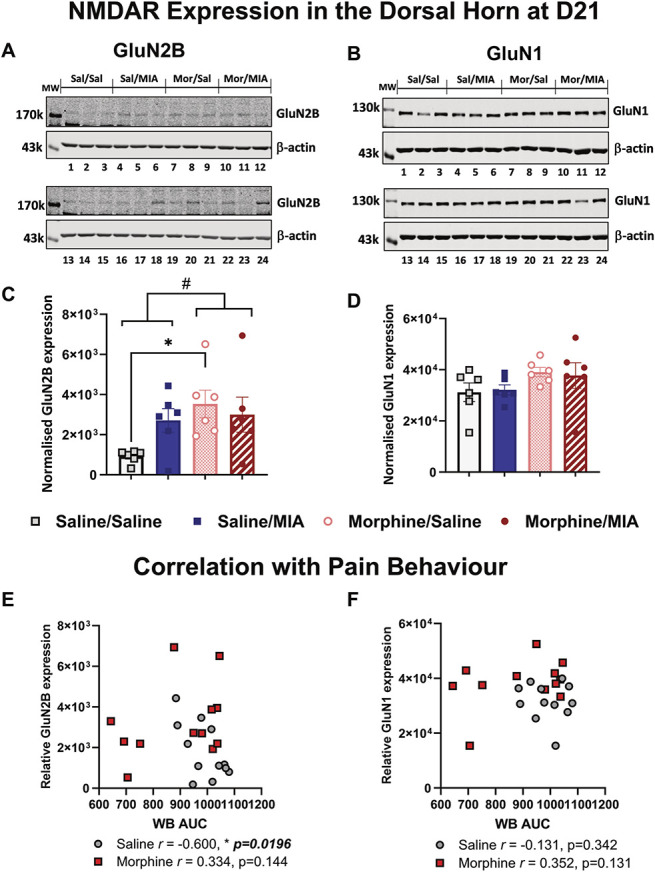
Sustained morphine exposure elevates spinal GluN2B expression at day 21. Expression of NMDAR subunits in ipsilateral spinal cord dorsal horn at D21 after intra-articular injection. GluN2B expression was elevated in the saline/MIA group when compared with saline/saline, but significance was not reached. A significant effect of drug treatment was observed, with GluN2B expression significantly higher in morphine-treated rats when compared with morphine-naïve groups (A and C). See supplementary Figure S5, http://links.lww.com/PR9/A224 for full blots. GluN1 expression in the dorsal horn of the spinal cord was comparable between the 4 groups (B and D, n = 6/group). In morphine naïve animals, weight-bearing asymmetry AUC was negatively correlated with GluN2B expression; this was not the case in the morphine-treated groups (E, data pooled by drug treatment and correlations determined through Pearson correlation coefficient *r*). Spinal expression of GluN2B was not correlated with PWTs (supplementary Figure S6C, http://links.lww.com/PR9/A224). NMDAR1 expression was not correlated with weight-bearing (F) or PWTs (supplementary Figure S6D, http://links.lww.com/PR9/A224). Data are presented as mean ± SEM and analysed via 2-way ANOVA with the Tukey multiple comparison *post hoc* test. #*P* < 0.05 vs saline-treated rats, *P < 0.05 vs saline/saline–treated rats. ANOVA, analysis of variance; AUC, area under the curve; MIA, monosodium iodoacetate; NMDAR, N-methyl-d-aspartate receptor; PWT, paw withdrawal threshold.

To assess whether changes in spinal NMDARs contribute to the augmented joint pain phenotype seen with sustained morphine treatment in this model, expressions of GluN2B (Fig. 5A) and GluN1 (Fig. 5B) were probed spinal lysates through Western blotting. In morphine-naïve rats, a trend towards increased expression of GluN2B was observed in the ipsilateral dorsal horn at D21 (Fig. 5A, C; saline/saline = 911 ± 130, saline/MIA = 2711 ± 588, morphine/saline = 3529 ± 688, morphine/MIA = 2995 ± 874, mean ± SEM), whereas morphine treatment significantly increased the expression of GluN2B in both groups (Fig. 5C, F_(1,20)_ = 5.27, *P* = 0.03). Spinal GluN1 expression was slightly altered by morphine treatment (Fig. 5B, D; F_(1,20)_ = 4.03, *P* = 0.06), but no significant differences were observed for any groups (saline/saline = 31179 ± 3631, saline/MIA = 32117 ± 1945, morphine/saline = 39076 ± 1791, morphine/MIA = 37736 ± 5009, mean ± SEM).

To determine whether spinal expression of NMDAR subunits was associated with pain behavior, relative expression levels of GluN2B (Fig. 5E) and GluN1 (Fig. 5F) were correlated with AUC data for WB asymmetry and PWTs. Dorsal horn GluN2B expression was negatively correlated with WB asymmetry (Pearson *r* = −0.60, *P* = 0.02), but not PWTs (Pearson *r* = 0.14, *P* = 0.33, supplementary Figure S5C, http://links.lww.com/PR9/A224), in morphine-naïve rats only (Fig. 5E). Dorsal horn expression of GluN1 was not correlated with either measure of pain behavior (Fig. 5F, supplementary Figure S5D, http://links.lww.com/PR9/A224).

## 4. Discussion

Mounting evidence suggests that prolonged prescription opioid treatment has significant long-term detrimental impacts on future pain experience, reducing or negating the benefits of surgical treatments on knee OA pain^9^ and indicating significant priming and/or plasticity of pain circuitry. Given the prevalence of both prescription opioid use and chronic OA pain, this represents a significant clinical burden, yet the underlying mechanisms remain poorly understood. In this study, we demonstrate an exacerbated pain phenotype in a model of sustained morphine exposure and OA pain in female rats, despite comparable joint pathology. We report significantly elevated spinal expression of astrocytic GFAP and neuronal GluN2B, but not microglial IBA1, in the dorsal horn following sustained morphine treatment. These morphine-driven increases were evident both in the presence and absence of the model of joint pain. Both glial cell signaling and NMDAR plasticity have established roles in spinal sensitization in models of chronic pain.^43,49^ Our data suggest that sustained morphine-induced changes in spinal GFAP-positive astrocytes and the expression of GluN2B-containing NMDARs prime the dorsal horn to incoming noxious inputs, resulting in the exacerbation of pain, despite comparable levels of joint damage.

### 4.1. Changes in dorsal horn GluN2B following sustained morphine exposure and relationships with osteoarthritis-like pain behavior

Spinal expression of GluN2B, but not GluN1, was elevated following sustained morphine treatment, both in the presence and absence of the model of joint pain. Spinal NMDARs have well-established roles in the development and induction of pain states, including inflammatory and neuropathic pain.^27^ GluN2B-containing NMDARs are predominantly expressed in the superficial dorsal horn, both presynaptically on central terminals of primary afferent neurons and postsynaptically on intrinsic neurons.^38^ In humans, GluN2B is coexpressed with substance P in sensory afferents, and expression is higher in female than in male individuals.^2^ GluN1 is expressed in most primary afferent subtypes^13^ and in a less restricted pattern throughout the grey matter of the dorsal horn.^38^ Presynaptic NMDARs are normally quiescent but play a role in the induction of chronic pain states.^13^

A trend towards elevated spinal GluN2B expression was observed in the model of OA pain in the absence of morphine treatment, but statistical significance was not reached. Rats treated with morphine in the absence of the model of OA pain did not exhibit significant changes in pain behavior, despite elevated spinal GluN2B expression. However, combining morphine treatment with the model of OA pain resulted in a significant exacerbation of pain behavior, potentially reflecting an interaction of increased dorsal horn expression of GluN2B and activation of GFAP-positive astrocytes.

Spinal expression of GluN2B was negatively correlated with changes in weight-bearing (but not mechanical thresholds) in morphine-naïve rats; however, this relationship was not evident in morphine-treated rats. This indicates dysregulation of gating of sensory inputs, GluN2B-mediated mechanisms, and pain behavior following sustained opioid treatment. Previous evidence demonstrated increased presynaptic NMDAR activity and potentiated glutamate release from primary afferent terminals following sustained opioid exposure, whilst NMDAR currents in the spinal dorsal horn were significantly reduced.^54,56^ The association between GluN2B and weight-bearing responses, but not distal mechanical thresholds, in morphine-naïve rats in this study may also point to a role of presynaptic GluN2B. Loss of β-arrestin–2 plays a significant role in the effects of MOR ligands on GluN2B-mediated excitability and directly affects GluN2B expression.^8^ It is also possible that chronic morphine exposure differentially regulates GluN2B phosphorylation in the presence and absence of a pain state. Phosphorylation of NMDARs alters channel properties, including opening, conductance, and desensitization and synaptic localization,^41^ and GluN2B possesses several phosphorylation sites and interacts with multiple kinases. A limitation of our study is that the synaptic localization of the changes in GluN2B expression and the phosphorylation state of this subunit were not determined, and probing these in future would be valuable. Taken collectively, these data and the existing literature support a mechanism by which sustained morphine treatment dysregulates the gating between painful inputs and GluN2B-mediated responses, priming the spinal dorsal horn to subsequent incoming sensory inputs, leading to exacerbated pain.

### 4.2. Changes in dorsal horn astrocytes, but not microglia, following sustained morphine exposure and relationships with osteoarthritis-like pain behavior

Spinal cord astrocytes have well-established contributions to central sensitization. Increased spinal GFAP expression is a marker for reactive astrogliosis and has been demonstrated in various models of chronic pain (see references in 18,24). Consistent with our previous studies using male rats,^6,49^ a trend towards increased dorsal horn GFAP expression was observed at D21 after MIA injection in female rats, suggesting astrogliosis may play a sex-independent role in spinal sensitization in this model. Sustained exposure to morphine produced a more pronounced increase in dorsal horn GFAP expression, both in the presence and absence of the model of joint pain. Of interest, rats receiving sustained morphine did not exhibit significant pain behavior, despite elevated GFAP expression, unless combined with the model of joint pain. This indicates spinal GFAP expression as a potential marker of opioid-induced priming. Repeated morphine exposure is associated with a sex-specific increase in GFAP labelling in some brain regions in rats,^4^ but an increase in spinal GFAP mRNA expression in both male and female mice.^29^ Crucially, astrocyte ablation impaired the induction and maintenance of opioid-induced hyperalgesia in this latter study, highlighting the critical role of astrogliosis in induction of pain states. Our data further support a role of spinal astrocytes in the exacerbation of OA-like pain behavior following sustained exposure to morphine.

In contrast to the marked effects of morphine treatment on GFAP-positive astrocytes, no significant changes in IBA1-positive microglial labelling were observed. Although the roles of microglia in mediating aberrant pain responses in models of chronic pain are well documented in male rodents, their role in females is subject to debate (see 32,33). Microglia are involved in opioid tolerance (see Ref. 53 for review), but their ablation does not alter opioid-induced hyperalgesia in either sex in mice or rats.^30^ Histological and RNA sequencing of purified spinal microglia in adult mice failed to provide evidence of MOR expression in these cells nor effects of chronic morphine on microglial phenotype.^11^ T cells have a known role in opioid analgesia in female mice,^47^ but their potential contribution to sustained opioid treatment–induced exacerbation of pain responses has yet to be explored.

### 4.3. Study design and sex

We focused this translational study on female rats to model the higher level of opioid prescribing and greater prevalence of OA pain observed clinically in female sex. The study design included assessment of 2 behavioral end points to provide additional mechanistic insight because changes in weight-bearing are generally considered to be peripherally driven with a contribution of central mechanisms, whereas changes in distal PWTs predominantly reflect central sensitization. Recapitulation of this widely used model of OA-like pain in female rats required some optimization. A higher dose of MIA (2 mg) was required to produce consistent, clinically relevant joint pathology, which was associated with significant WB asymmetry up to D14, but not D21. This observation is consistent with previous reports in female rats^16,40^ and differs from the sustained WB asymmetry widely reported in male rats.^10,21^ The model alone did not produce sustained lowering of ipsilateral PWTs in female rats, consistent with previous reports,^40^ but in marked contrast to male rats.^10^ We speculate that this could be due to sex differences in learning or heightened stress-induced analgesia in female sex due to the repeated handling, dosing, and testing in our experimental paradigm. Differences in pain phenotypes exhibited between sexes of the same strain of rat in this model highlights the importance of assessing multiple pain behaviors in both sexes for mechanistic interrogation.

## 5. Conclusion

This study reports that sustained morphine treatment increases dorsal horn expression of markers for astrocytes, but not microglia, and elevates GluN2B expression. Based on the established critical roles of both astrocytes and NMDARs in central sensitization, it is likely that these changes in cellular and receptor function primes the dorsal horn to incoming noxious inputs, resulting in the exacerbation of joint pain behavior despite comparable levels of joint damage. Our back-translation of the effects of opioid exposure on future pain responses provides a new experimental approach to interrogate the underlying peripheral, spinal, and supraspinal mechanisms that contribute to opioid-induced hyperalgesia and its consequences on treatment outcomes for chronic OA pain.

## Disclosures

The authors have no conflict of interest to declare.

## Appendix A. Supplemental digital content

Supplemental digital content associated with this article can be found online at http://links.lww.com/PR9/A224.

## Supplementary Material

SUPPLEMENTARY MATERIAL
