# Supplementary material for: Sustained morphine exposure alters spinal NMDA receptor and astrocyte expression and exacerbates chronic pain behavior in female rats
Source: Pain Rep. 2024 Mar 12;9(2):e1145. doi: 10.1097/PR9.0000000000001145 (PMC10936964; doi:10.1097/PR9.0000000000001145)
Supplement: SUPPLEMENTARY MATERIAL [file painreports-9-e1145-s001.pdf]

# Supplementary Materials

# Sustained morphine exposure alters spinal NMDA receptor and astrocyte expression and exacerbates chronic pain behaviour in female rats

Sara V. Gonçalves[a,b,+], Stephen G. Woodhams[a,b,+], Li Li[a,b], Gareth J. Hathway[a,b,c], Victoria Chapman[a,b,c] *

[a] Pain Centre Versus Arthritis, University of Nottingham, Medical School, Queen's Medical Centre, Nottingham, [b] School of Life Sciences, Medical School, Queen's Medical Centre, Nottingham,
[c] NIHR Nottingham Biomedical Research Centre, University of Nottingham, Nottingham, United Kingdom

+ Joint first authors Sara Gonçalves & Stephen G Woodhams contributed equally to this work

* Corresponding author. Address: E177, School of Life Sciences, Medical School, Queen's Medical Centre, Nottingham, United Kingdom, NG7 2UH. Tel.: +44115 82 30136; Fax: +44115 82 30142. E-mail address: Victoria.chapman@nottingham.ac.uk.

**Running Title: Morphine induced spinal plasticity and pain**

**Disclosures:** This work was supported by Arthritis Research United Kingdom [grant number 20777]; and the Medical Research Council research [grant number MR/W019663/1]. The authors declare no conflicts of interest.

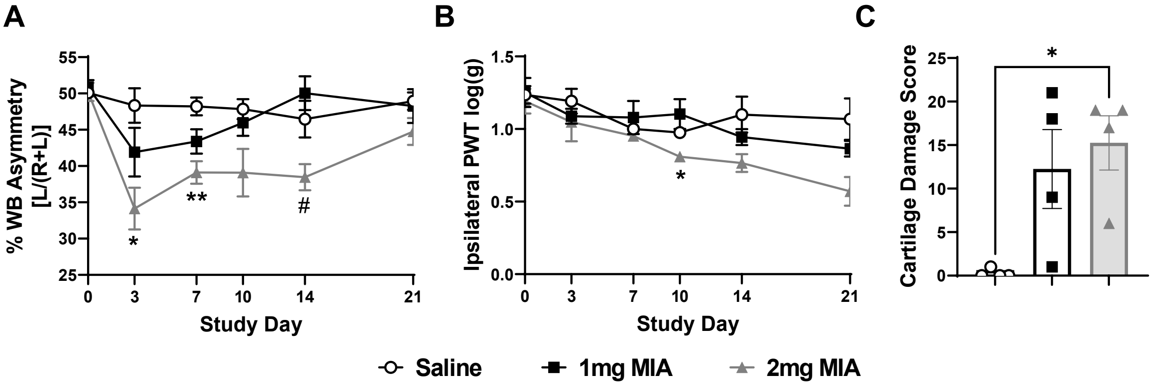


**Figure S1 – OA-like pain and knee pathology in female rats induced with different doses of MIA.** Female Sprague Dawley rats received intra-articular injection of either 1mg/50µl MIA (■), 2mg/50µl MIA (●) or 50µl saline (○) into the left knee at day 0 (n=4/group). Rats injected with 2mg MIA exhibited higher weight bearing asymmetry (**A**) compared to those injected with 1mg MIA. Injection of 2mg MIA induced a slight decrease in PWT (**B**), but only at later time points. Injection of 1mg MIA did not significantly reduce PWTs compared to injection of saline. Chondropathy was observed in both MIA-injected groups, but only 2mg MIA induced significant knee pathology when compared with saline controls.

Data are presented as mean±SEM. *p=0.5, **p<0.01, versus saline; #p<0.05 versus 1mg MIA. RM 2-way ANOVA with Tukey's post-hoc testing. ANOVA, analysis of variance; MIA, monosodium iodoacetate; OA, osteoarthritis; PWTs, paw withdrawal thresholds.

| ***Protein loading per sample*** | ***Target Protein*** | ***Suppliers*** | ***Source*** | ***Dilution for WB*** |
|---|---|---|---|---|
| 30μg | IBA1 | Novus/NB100-1028 | Goat | 1:1000 |
| 5~10μg | GFAP | Dako/Z0334 | rabbit | 1:2000 |
| 30μg | NMDAR1 | abcam/ab109182 | rabbit | 1:1000 |
| 40μg | NMDAR2b | abcam/ab65783 | rabbit | 1:1000 |
| | Beta-actin | Sigma-Aldrich /a5441 | mouse | 1:2000 |

**Table S1 – Protein isolation and Western blotting.**

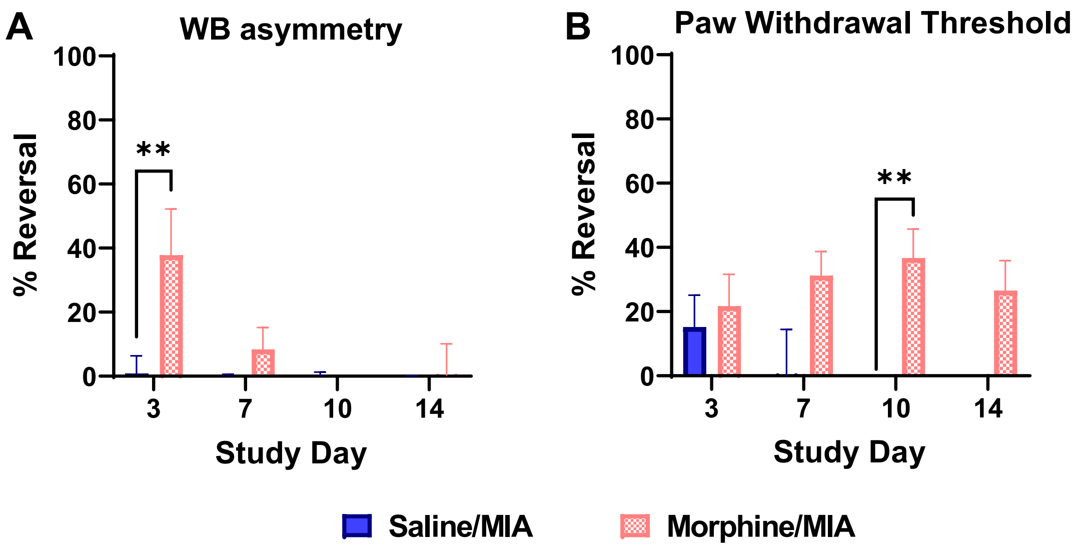


**Figure S2 –Acute analgesic effects of morphine versus saline administration in the MIA model of OA-like pain in female rats.** See figure 1A for timeline. To evaluate the acute analgesic effects of morphine treatment, pain behaviour was assessed 1hr after morning systemic injection. Morphine acutely reversed weight-bearing asymmetry (**A**) on day 3, but not at any later time points. No significant effect of drug treatment was observed, but there was a significant effect of time, and interaction between time and drug treatment (treatment: $F_{(1,9)}$=2.37, p=0.16; time: $F_{(1.7,15.3)}$=7.3, p=0.001; treatment x time: $F_{(3,27)}$=5.5, p=0.004, 2-way ANOVA). Šídák's multiple comparisons test revealed a significant difference between saline/MIA and morphine/MIA rats on day 3 only (p<0.01). Morphine also produced an acute, partial reversal of MIA-induced reductions in ipsilateral PWT (**B**), across the entire duration of the study (treatment: $F_{(1,10)}$=23.6, p=0.0007; time: $F_{(3,30)}$=0.27, p=0.85; treatment x time: $F_{(3,30)}$=1.5, p=0.25, 2-way ANOVA). Šídák's multiple comparisons test revealed a significant difference between saline/MIA and morphine/MIA rats on day 10 only (p<0.01). Data are presented as mean±SEM.

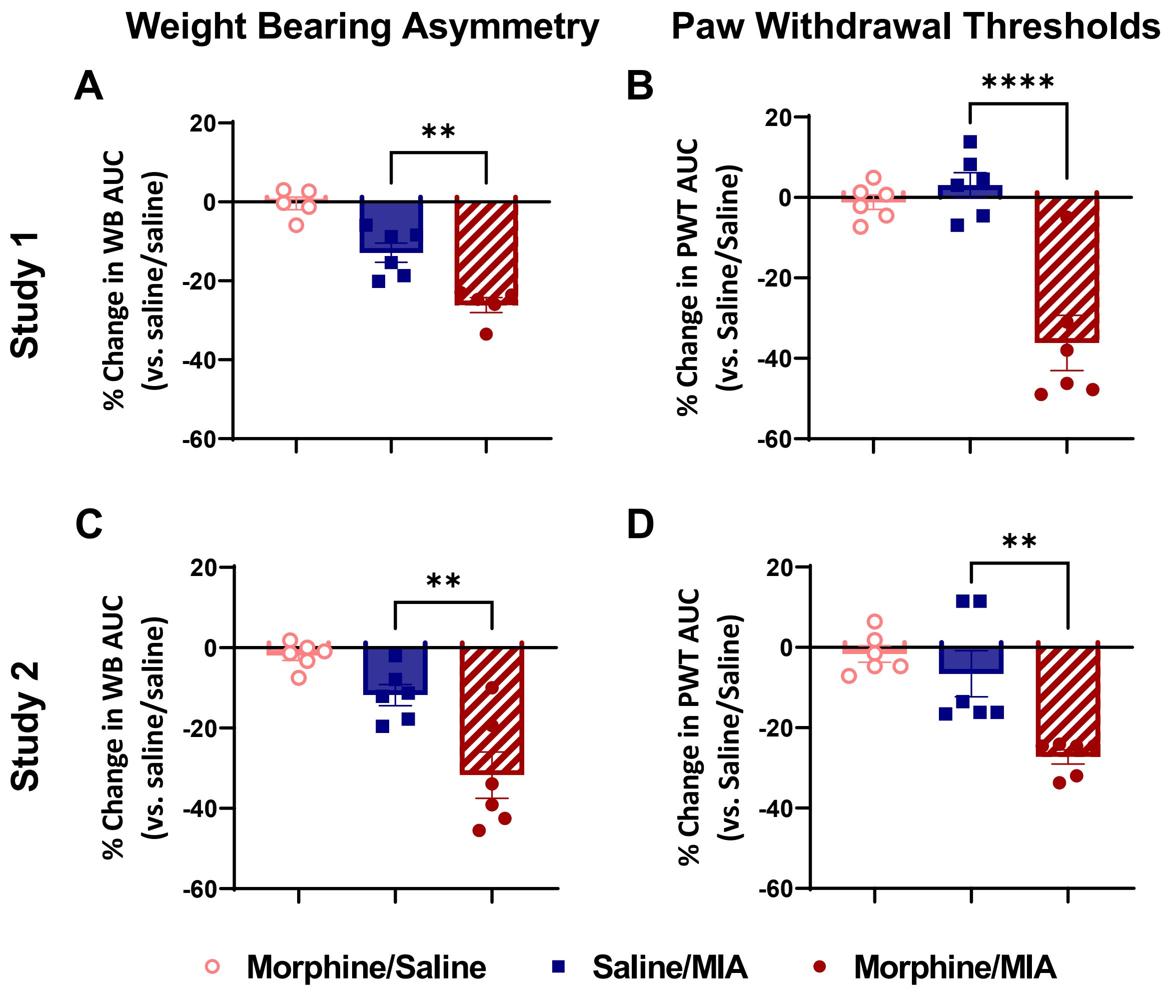


**Figure S3 – Sustained exposure to morphine exacerbates weight bearing asymmetry and lowers PWTs in female rats to a similar degree 2 weeks after MIA injection in separate cohorts of animals.** Area under the curve (AUC) data generated from time course data in figure 2, and expressed as % change compared to saline/saline. Unilateral intra-articular injection of 2mg MIA produced significant weight bearing asymmetry up to D14 in both study 1 (**A;** $F_{(2,13)}$=35.76, p<0.0001) and study 2 (**C;** $F_{(2,15)}$=16.66, p=0.0002), but no significant reduction in PWTs in either study (**B** & **D**). Sustained morphine exposure significantly exacerbated MIA-induced weightbearing asymmetry at D14 after MIA-injection in both study 1 (**A**) and study 2 (**C**), and substantially lowered PWTs (**B:** $F_{(2,15)}$=23.04, p<0.0001; **D:** $F_{(2,15)}$=13.78, p=0.0004). Sustained morphine exposure in the absence of the MIA model had no effect on either weight bearing or PWTs. These data confirm that sustained morphine exposure reproducibly exacerbates OA-like pain in the MIA model in female rats. Data are presented as mean ± SEM, n=6/group. **p<0.01, ****p<0.0001. One-way ANOVA with Tukey's *post-hoc* testing.

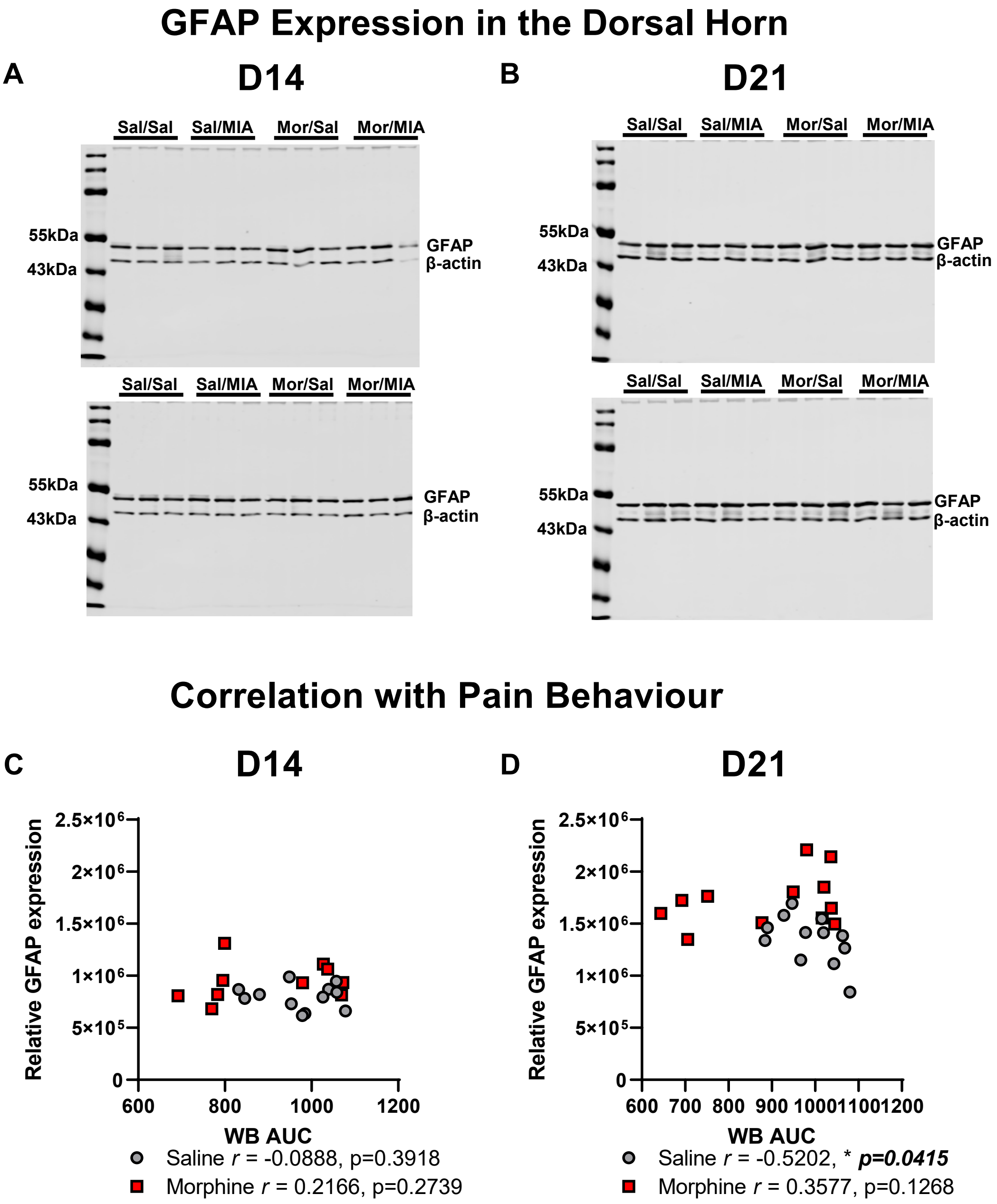


**Figure S4 – GFAP expression in the spinal cord dorsal horn in a model of sustained opioid exposure and OA-like pain.** Expanded full-length western blots from the panels shown in figure 4, depicting GFAP expression at D14 (**A**) and D21 (**B**) after intra-articular injection. No significant correlation was observed between weightbearing asymmetry AUC and spinal GFAP at D14 in study 1 (**C**). In contrast, spinal GFAP expression was significantly negatively correlated with weight bearing asymmetry at D21 in study 2 (**D**), but only in morphine-naïve rats.

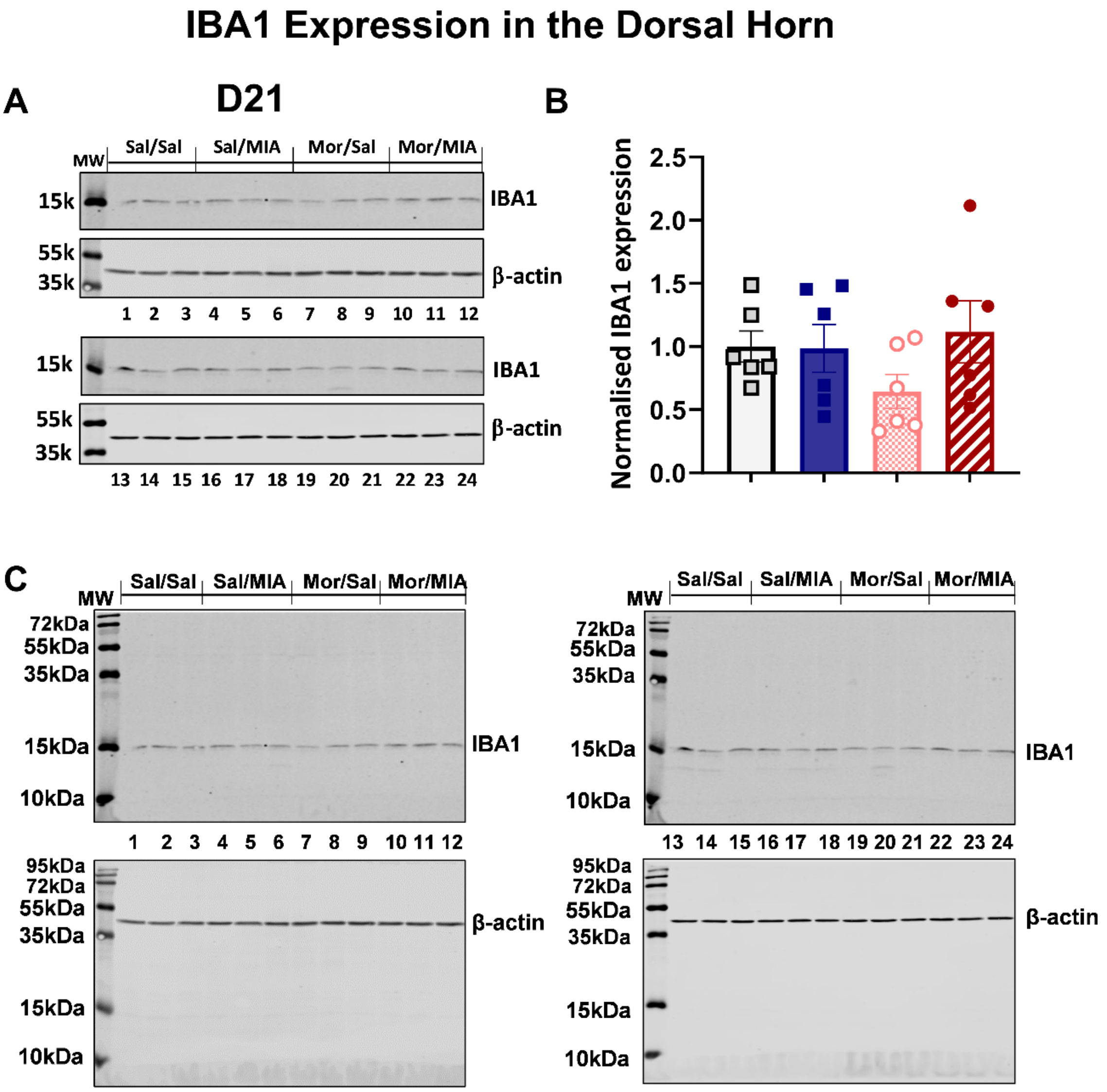


**Figure S5 – IBA1 expression in the spinal cord dorsal horn is unaltered at D21 in a model of sustained opioid exposure and OA-like pain.**

Spinal ipsilateral dorsal horn expression of IBA1 was similar in across all treatment groups at D21 after intra-articular injection (**A**, *n*=6/group). Quantification via densitometry showed no significant differences in IBA1 protein expression levels, suggesting microglia may not play a significant role in maintenance of chronic pain or the effects of sustained opioid exposure in this model (**B**). Expanded full-length western blots from panel **A** (**C**).

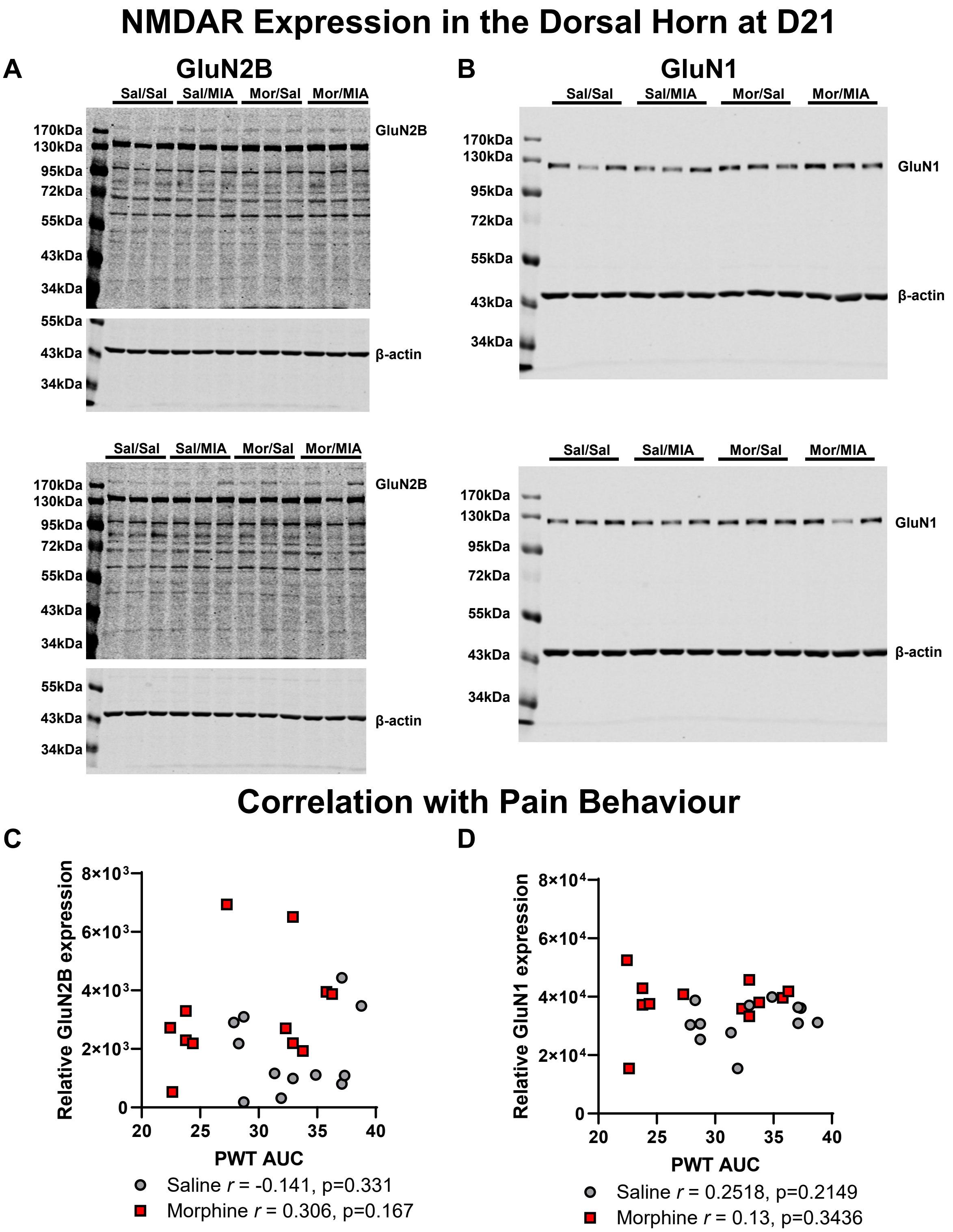


**Figure S6 – NMDAR expression in the spinal cord dorsal horn in a model of sustained opioid exposure and OA-like pain.** Expanded full-length western blots from the panels shown in figure 5, depicting spinal GluN2B (**A**) and GluN1 (**B**) expression after intra-articular injection. Spinal expression of GluN2B (**C**) or GluN1 (**D**) was not significantly correlated with PWT AUC at D21 in study 2. Correlations assessed via Pearson's correlation coefficient r.
